# A Bifunctional Nanosilver-Reduced Graphene Oxide Nanocomposite for Label-Free Electrochemical Immunosensing

**DOI:** 10.3389/fchem.2021.631571

**Published:** 2021-04-28

**Authors:** Supakeit Chanarsa, Jaroon Jakmunee, Kontad Ounnunkad

**Affiliations:** ^1^Department of Chemistry and Center of Excellence for Innovation in Chemistry, Faculty of Science, Chiang Mai University, Chiang Mai, Thailand; ^2^The Graduate School, Chiang Mai University, Chiang Mai, Thailand; ^3^Research Center on Chemistry for Development of Health Promoting Products From Northern Resources, Chiang Mai University, Chiang Mai, Thailand; ^4^Center of Excellence in Materials Science and Technology, Chiang Mai University, Chiang Mai, Thailand

**Keywords:** silver nanoparticles, reduced graphene oxide, electrochemistry, immunosensor, Immunoglobulin G, screen-printed carbon electrode

## Abstract

A bi-functional material based on silver nanoparticles (AgNPs)-reduced graphene oxide (rGO) composite for both electrode modification and signal generation is successfully synthesized for use in the construction of a label-free electrochemical immunosensor. An AgNPs/rGO nanocomposite is prepared by a one-pot wet chemical process. The AgNPs/rGO composite dispersion is simply cast on a screen-printed carbon electrode (SPCE) to fabricate the electrochemical immunosensor. It possesses a sufficient conductivity/electroreactivity and improves the electrode reactivity of SPCE. Moreover, the material can generate an analytical response due to the formation of immunocomplexes for detection of human immunoglobulin G (IgG), a model biomarker. Based on electrochemical stripping of AgNPs, the material reveals signal amplification without external redox molecules/probes. Under optimized conditions, the square wave voltammetric peak current is responded to the logarithm of IgG concentration in two wide linear ranges from 1 to 50 pg.ml^−1^ and 0.05 to 50 ng.ml^−1^, and the limit of detection (LOD) is estimated to be 0.86 pg.ml^−1^. The proposed immunosensor displays satisfactory sensitivity and selectivity. Importantly, detection of IgG in human serum using the immunosensor shows satisfactory accuracy, suggesting that the immunosensor possesses a huge potential for further development in clinical diagnosis.

## Introduction

Development of new strategies and sensitive, selective, and low-cost devices for detection of clinically predictive bioindicators in the human body with good accuracy and precision has received considerable attention (Reanpang et al., 2015; Justino et al., 2016; Sharafeldin et al., 2017). These devices have been extensively studied for screening, monitoring, and diagnosing diseases and virus infections (Gug et al., 2019; Metkar and Girigoswami, 2019; Roointan et al., 2019; Sadighbayan et al., 2019; Farzin et al., 2020; Menon et al., 2020). In particular, the electrochemical immunosensing strategy is one of the most studied methods, because it combines advantages of antibodies via immunoreaction with high specificity, which gives high sensitivity and fast response (Luppa et al., 2001; Rama and Costa-García, 2016; Putnin et al., 2018). Moreover, for applications in point-of-care (POC) clinical testing, it can be miniaturized and developed for *in situ* detection and real-time assay (Janmanee et al., 2012; Thunkhamrak et al., 2017, 2020; Pothipor et al., 2018; Gao H. et al., 2019; Jofre et al., 2020; Regiart et al., 2020; Upan et al., 2020). These reasons make an immunosensor very attractive for applications in assays of clinically relevant analytes (Luppa et al., 2001; Rama and Costa-García, 2016).

Many reports showed uses of sandwich-type electrochemical immunosensors to detect various kinds of biomarker proteins popularly due to high sensitivity and specificity (Huang et al., 2015; Miao et al., 2019; Pei et al., 2019a; Zhang C. et al., 2019; Zhang S. et al., 2019; Awan et al., 2020; Li et al., 2020a; Pourmadadi et al., 2020). Although the immunosensors show high performance in the detection of target analytes, some devices present limits of detection (LOD) comparable to those of label-free configuration, which has less complexity. Chen et al. (2020) reported that a sandwich-type sensor with a dual-signal amplification element mechanism used in the detection of carcinoembryonic antigen (CEA) obtained an LOD of 0.032 pg ml^−1^. Additionally, signal amplification using high-surface area graphene (GP) nanocomposites presented an LOD value of 100 pg ml^−1^ (Chen et al., 2013), and Ag/Au nanoparticles (NPs)/GP provided an LOD value of 8 pg ml^−1^ (Huang et al., 2015). With more complexity using glucose oxidase-encapsulated gold (Au) hollow microspheres and pH meter readout, an electrochemical immunosensor provided an LOD value of 62 pg ml^−1^ (Jiang et al., 2018), while a label-free immunosensor based on Bi_2_MoO_6_ nano-tremella (NT) showed an LOD value of 0.3 pg ml^−1^ for CEA assay (Wang et al., 2020). Recently, many reports have shown insignificant improvement in the LOD value for the detection. Deng et al. (2020), Li et al. (2020b), and Shen et al. (2020) demonstrated sandwich-type immunosensors with LOD values of 0.48, 0.5, and 5 pg.ml^−1^, respectively. Employing other techniques, together with electrochemical process, in sandwich-type immunosensing, such as electrochemiluminescence (LOD of 3 pg ml^−1^) (Huang et al., 2018) and photoelectrochemical format (LOD of 0.468 pg ml^−1^) (Liu et al., 2020), also offers LOD values comparable with those of the label-free configuration. Moreover, a photoelectrochemical immunodevice with a 3D origami platform was used for detection of CEA with a lower LOD value of 0.3 pg ml^−1^ (Ge et al., 2015). For detection of a target protein, new and highly complicated methods would not show significantly better performance compared with the label-free immunosensors. Furthermore, a label-free sensor using electrochemiluminescence response revealed no significant difference in LOD value (0.23 pg ml^−1^) (Zhang et al., 2020). Therefore, the label-free electrochemical immunodevice is interesting due to its low cost, easy to operate instrumentation, and no time consumption. This device can reduce chemical consumption and complexity such as fabrication steps.

There are excellent nanomaterials such as graphene oxide (GO) (Jumpathong et al., 2016), Au@Bi_2_MoO_6_ NTs (Wang et al., 2020), Pt–Au alloy nanotube array (Tao et al., 2011), Au–Pt nanostructures (Jia et al., 2015), Au@Pd/Ag NPs and amination GP (Li N. et al., 2014), AuNPs and poly(amidoamine)-MWCNTs-chitosan nanocomposite (Dong et al., 2013), AuPd@Au nanocrystals (Wang et al., 2018b), Au@Pt nanocrystals (Wang et al., 2018c), AuNPs/Zn/Ni–ZIF-8-800@GP composites (Hu et al., 2019), PdCu tripod functionalized porous GP (Tan et al., 2020), Cu_3_Pt nanoframes (Wang et al., 2018a), Pd NPs@3D MoS_x_ (Gao Z. et al., 2019), 3D PtCu nanoframes (Chen et al., 2019), Ag/MoS_2_/rGO nanocomposites (Wang et al., 2018), AuPdCu NPs/N-doped GP quantum dots functionalized polymer nanospheres (Yan et al., 2018), Pd NPs functionalized MoS_2_/NiCo heterostructures (Ding et al., 2020), Au NPs/MoS_2_-GP aerogels composite (Xu et al., 2020), AuNPs–PtNPs–MOFs (Zhao et al., 2019), and PtPd NCs@MoS_2_ nanoenzymes (Tan et al., 2019), which have been employed in the construction of label-free electrochemical immunosensors. Their label-free immunosensors are demonstrated with additional chemicals or redox probes such as H_2_O_2_ (Tao et al., 2011; Wang et al., 2018, Leng et al., 2011; Yan et al., 2018; Chen et al., 2019; Gao Z. et al., 2019; Pei et al., 2019a,b; Tan et al., 2019; Zhao et al., 2019; Ding et al., 2020), O_2_ (Wang et al., 2018a,c; Chen et al., 2019), methyl orange (Sun et al., 2019), and [Fe(CN)_6_]^4−/3−^ (Dong et al., 2013; Li R. et al., 2014; Liu et al., 2015; Han et al., 2017; Hu et al., 2019; Xu et al., 2020) to obtain signal amplification via electrocatalytic reductions and charge transfer reactions. Moreover, the nanomaterials can improve the conductivity and electrochemical reactivity of an electrode surface. Han et al. reported an immunosensor based on rGO/Ag NPs composites in the detection of target protein by signal amplification via blockage of the electron transfer process in [Fe(CN)_6_]^4−/3−^ due to the amount of immunocomplex (Han et al., 2017). The composites can immobilize antibodies without covalent bonding. Interestingly, some nanomaterials, namely, AgPt nanorings/reduced GO (Wang et al., 2018a) and Ag–GO nanocomposites (Wu et al., 2013), can amplify analytical responses in label-free immunosensing with no addition of chemicals. The responses are from the redox processes of surface-confined AgPt nanorings/reduced GO and Ag–GO, which are restricted by immunocomplexes formed on the electrode surface. Therefore, development of label-free immunosensors with no complication is challenging. These immunosensors require versatile materials for device construction, which can improve electrode reactivity and protein immobilization without coupling agents and can perform with signal generation.

Taking into account the need for advancements in the diagnosis of target biomarker proteins, this study presents the development of a simple, sensitive, cost-effective, and label-free electrochemical immunosensor for the detection of immunoglobulin G (IgG) as a model protein in human serum. Compared with that in the study of Han et al. (2017), the strategy used in this study requires no other redox probes for label-free assay. Wu et al. demonstrated a label-free Ag–GO-based immunosensor, which required a step for the deposition of an Au film (Wu et al., 2013). Although AgPt nanorings/reduced GO offered very low LOD, the sensor consumed an expensive reagent (Wang et al., 2018a). Additionally, the label-free electrochemical determination of IgG in our study was demonstrated by the proof-of-concept development of sensor-based signal-amplifying AgNPs on rGO. A bifunctional material based on silver nanoparticles (AgNPs)-reduced graphene oxides (rGO) was employed for both electrode modification and signal generation. The biocompatibility and electrochemical properties of screen-printed carbon electrodes (SPCEs) were improved by modification with such AgNPs-rGO. The reduction in electrochemical stripping response of AgNPs is proportional to the amount of target IgG in the electrode. Under optimized conditions, our proposed immunosensor obtained satisfactory sensitivity and selectivity, two wide dynamic ranges, and low LOD of 0.86 pg.ml^−1^. The immunosensor was successfully examined for the detection of IgG in human serum with good recoveries.

## Experiment

### Chemicals and Materials

Anti-human IgG (Fab specific) antibody (anti-IgG, 5.5 mg ml^−1^) produced in goats, dopamine hydrochloride (DA, 99.5%), graphite powder (synthetic, size <20 μm), IgG from human serum (IgG, 4.8 mg ml^−1^, ≥95%), phosphate-buffered saline (PBS) tablets (pH 7.4), myoglobin from the human heart (Mb, 2.4 mg ml ^−1^, ≥95%), and human serum from a male (AB plasma, United States origin, lot: SLBS6544) were purchased from Sigma Aldrich (Singapore). Potassium ferricyanide (K_3_[Fe(CN)]_6_, 98.5%) (), sulfuric acid (H_2_SO_4_, 96%), potassium nitrate (KNO_3_, 99%), hydrochloric acid (HCl, 37%), and ethanol (C_2_H_6_O, 95%) were obtained from Lab Scan (Gliwice, Poland). L(+)-ascorbic acid (C_6_H_8_O_6_, 99.7%), sodium di-hydrogen phosphate dehydrate (NaH_2_PO_4_2H_2_O, 98.5%), and nitric acid (HNO_3_, 65%) were ordered from Merck (Darmstadt, Germany). Bovine serum albumin (BSA, 98%) was obtained from Merck (Germany). Di-sodium hydrogen phosphate dehydrate (Na_2_HPO_4_2H_2_O, 99%), glucose (C_6_H_12_O_6_, 99%), hydrogen peroxide (H_2_O_2_, 50%), potassium permanganate (KMnO_4_, 99%), silver nitrate (AgNO_3_, 99.8–100.5%), and uric acid (C_5_H_4_O_3_N_4_, ≥99%) were purchased from Scharlau (Barcelona, Spain), Fluka (Buchs, Switzerland), AJAX (NSW, Australia), Carlo Erba (Cornaredo, MI, Italy), BDH Chemical Ltd. (Poole, England), and Sigma-Aldrich (St. Louis, United States), respectively. Interleukin-15 (IL-15, lot: 2381730, ≥ 98%) was purchased from Millipore (Burlington, MA, United States). Deionized water was used throughout this study. Sodium citrate (Na_3_C_6_H_5_O_7_, 99%) was purchased from Merck (Germany).

### Synthesis of AgNPs/rGO Composite

Graphene oxide (GO) powder achieved from the modified Hummers process (Hummers and Offeman, 1958; Pothipor et al., 2015) was employed for the synthesis of an AgNPs/rGO composite (Han et al., 2017). Briefly, GO (25 mg) and AgNO_3_ (15 mg) were mixed in DI water (50 ml) under stirring at 95°C and then Na_3_C_6_H_5_O_7_ (25 mg) was added into the mixture, which was continuously stirred for 1 h. After stirring, the AgNPs/rGO composite obtained was washed with distilled water by centrifugation at 9,500 rpm for 25 min. The washing process was repeated for a few times. Finally, the AgNPs/rGO composite powder was dried at 60°C overnight.

### Apparatus and Instrumentation

Scanning electron microscopy (SEM) photographs of the electrode surfaces were recorded using a JSM-6335F field emission scanning electron microscope (JEOL, Tokyo, Japan), and transmission electron microscopy (TEM) images were obtained using a JEM 2010 transmission electron microscope (JEOL, Tokyo, Japan). Raman spectra were recorded using a T64000 Raman spectrometer (Horiba Jobin Yvon, Villeneuve d'Ascq, France). All electrochemical experiments were carried out using a three-electrode electrochemical cell configuration. A platinum (Pt) wire (Nilaco Co. Ltd., Tokyo, Japan), a silver/silver chloride (Ag/AgCl, 3M NaCl) from BASi (West Lafayette, United States), and a SPCE were employed as counter, reference, and working electrodes, respectively. The SPCEs were prepared following the optimal condition described in previous reports (Reanpang et al., 2015). To obtain modified SPCEs, 4-μl droplets of 1.5 mg.ml^−1^ GO or AgNPs/rGO dispersion solution were added onto the plasma-cleaned SPCEs (Rama and Costa-García, 2016; Jiang et al., 2018), and then the electrodes were dried with air. Cyclic voltammetry (CV) and square wave voltammetry (SWV) measurements were carried out using an Emstat 3 potentiostat (PalmSens, Houten, the Netherlands). Electrochemical impedance spectroscopy (EIS) measurement was performed using a PGSTAT302N Autolab potentiostat (Metrohm, Barendrecht, the Netherlands).

### Fabrication of the Immunosensor

**Scheme 1:**
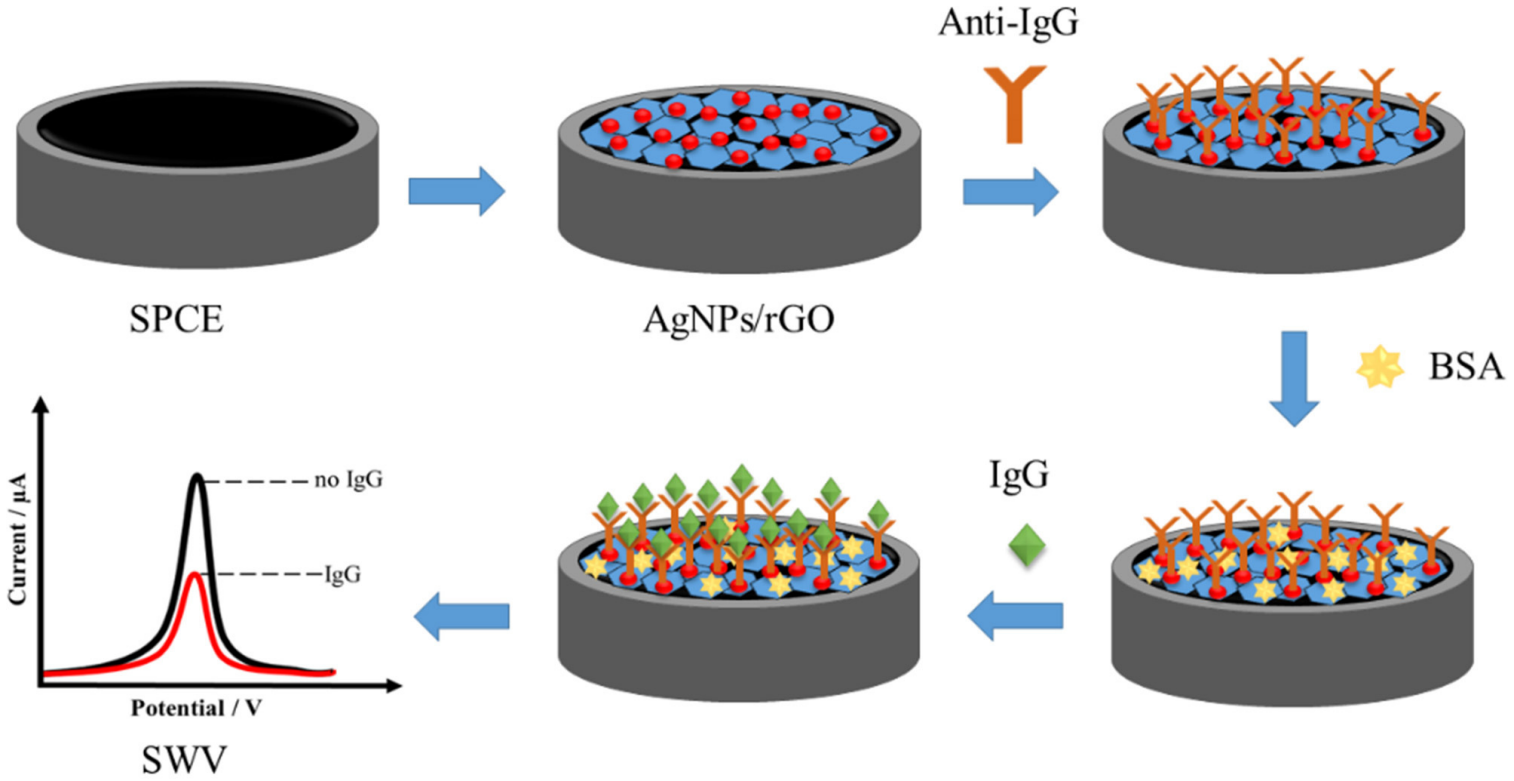
Fabrication of AgNP/rGO-based electrochemical immunosensor.

Scheme 1 shows the fabrication of the label-free AgNP/rGO-based electrochemical immunosensor. First, the droplet of 1.5 mg.ml^−1^ AgNPs/rGO dispersion solution was added onto the plasma-treated SPCE (Jumpathong et al., 2016; Putnin et al., 2018), and then the electrode was dried with air. Then, the AgNPs/rGO-coated working electrode was washed with a 0.01 M PBS buffer (pH 7.4) solution several times. The modified electrode was incubated with a 7.5 μl of 50 μg.ml^−1^ anti-IgG antibody solution for 40 min at room temperature. After washing with 0.01 M PBS buffer (pH 7.4) several times, the electrode was incubated with 7.5 μl of 1 wt % BSA solution for 40 min to eliminate and block non-specific binding of other substances on the electrode surface. Finally, the electrode was washed again with PBS buffer several times, and then for immunoassay, the electrodes were incubated with a 7.5 μl solution at different concentrations of IgG (1, 2.5, 5, 10, 25, 50, 100, 250, 500, 1,000, 2,500, 5,000, 10,000, 25,000, and 50,000 pg ml^−1^) for 40 min at room temperature in order to construct the calibration curve. Consequently, electrodes with different IgG amounts were washed gently with the PBS buffer several times to remove unbound IgG molecules. The square wave voltammetric scan of the immunosensing electrode before and after incubation with each IgG solution was taken at 0–0.35 V (vs. Ag/AgCl) in 0.1 M PB (pH 7.4).

## Results and Discussion

### Properties of Ag/rGO Composite

**Figure 1:**
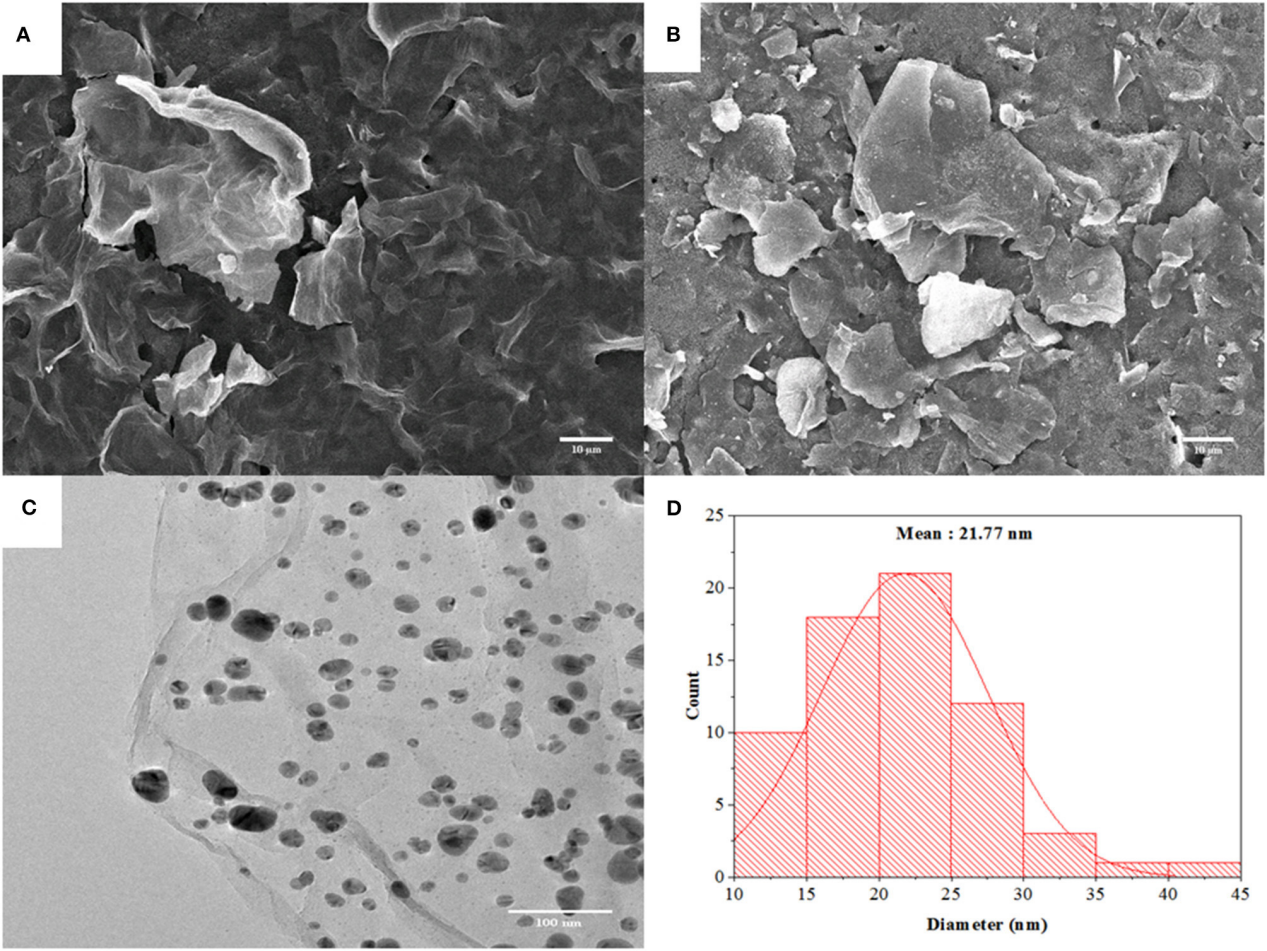
SEM images of GO **(A)** and AgNPs/rGO **(B)** coated on SPCEs. A TEM image **(C)** of AgNPs/rGO and a particle size distribution profile **(D)** of AgNPs on rGO sheets.

**Figure 2:**
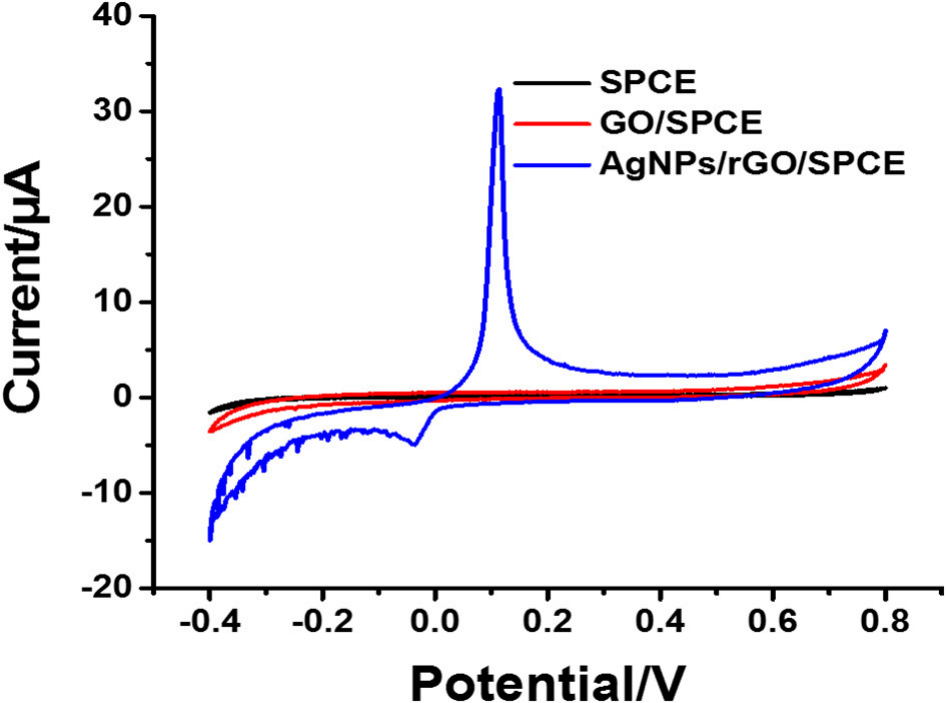
CV results of bare SPCE, and GO and AgNPs/rGO-modified SPCEs in contact with PB (pH 7.4).

The morphology of GO and AgNPs/rGO modified on SPCEs was investigated by SEM observation. As shown in Figure 1A, GO reveals a thin wrinkled blanket-like structure with some corrugations covering the whole SPCE surface. Compared with Figures 1A,B shows a large irregular shape of rGO nanosheets. The particle surface of rGO is smooth while that of AgNPs could not be observed. Figures 1C,D present a TEM image of an AgNPs/rGO sample, which is fully exfoliated into an individual sheet with smooth surface and corresponding particle size distribution profile. The anchored AgNPs are uniformly distributed on the surface of the rGO. The result indicates a strong interaction between AgNPs and the compatible rGO support, which offers a sufficient number of chemically active sites for deposition of well-dispersed AgNPs (Wu et al., 2013; Han et al., 2017). The average size for decorating AgNPs is estimated to be 21.77 nm. High dispersibility of AgNPs on rGO nanosheets would give high surface area, resulting in good reactivity of the modified SPCE and a large amount of loaded antibodies. This would offer a great sensing performance. Moreover, observable background current in CV (see Figure 2) is clearly increased due to increased surface area of the electrode by rGO modification together with introduction of AgNPs deposition (Nossol et al., 2014). Raman spectroscopy has been performed recently to identify the electronic characteristics and structure of graphene-based materials, defect structures, and disorder (Liu et al., 2012; Das et al., 2014). In Supplementary Figure 1, Raman spectra of SPCE, GO, and AgNPs/rGO present two major characteristic peaks, D band and G band, which represent the symmetric A_1g_ breathing mode and the E_2g_ mode of *sp*^2^ carbon atoms, respectively (Das et al., 2014). In addition, the 2D peak represents the second order of zone-boundary phonons and varies with the number of layers in graphene flakes (Liu et al., 2012). In this study, the D, G, and 2D bands of GO sitting on SPCE are located at 1,347, 1,584, and 2,690 cm^−1^, respectively. After reduction, there are no significant changes in peak positions for all bands. Changes in the relative intensity of the D and G bands (*I*_D_/*I*_G_) display alterations in the electronic conjugation state within the GO sheets during reduction. Additionally, the *I*_D_/*I*_G_ value of AgNPs/rGO is almost the same as that of GO, implying that reduction caused no additional structural defects during the one-pot synthesis of AgNPs/rGO (Das et al., 2014; Han et al., 2017).

### Electrochemical Study of AgNPs/rGO Composite

**Figure 3:**
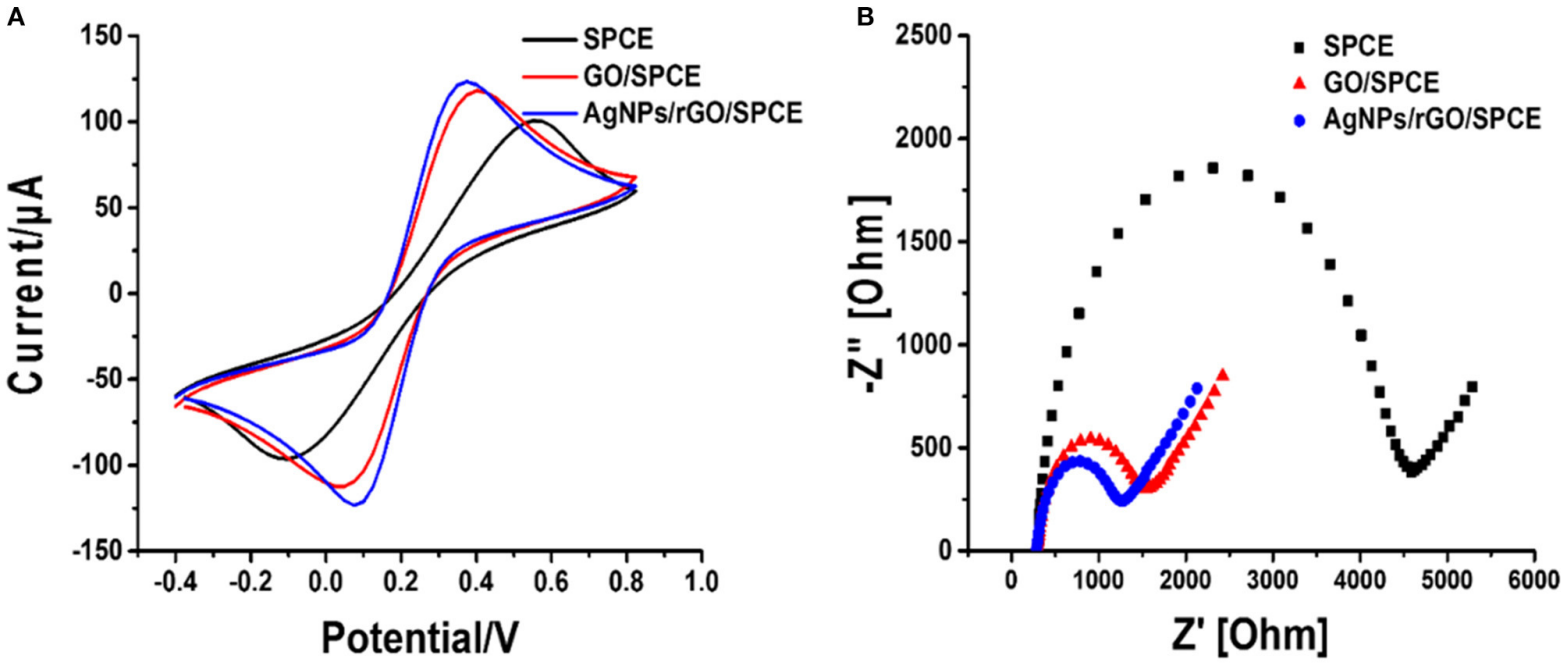
**(A)** CV curves at a scan rate of 100 mV s^−1^ and **(B)** Nyquist plots of bare SPCE, and GO- and AgNP/rGO-modified SPCEs in contact with 0.10 M PB (pH 7.4) containing 5 mM K_3_[Fe(CN)_6_]/K_4_[Fe(CN)_6_].

The electrochemical characteristics and performance of the modified SPCEs in 0.10 M PB (pH 7.4) at a scan rate of 100 mV.s^−1^ were investigated by CV. CV is a capable method for probing an electrochemical process in electrode modification. The CV results of bare SPCE and SPCEs modified with different materials (GO and AgNPs/rGO) in contact with 0.10 M PB (pH 7.4) are displayed in Figure 2. The curves of the based SPCE (black line) and GO-modified SPCEs (brown line) electrode show no oxidation peak current because of the absence of redox species, namely, redox probe (AgNPs). However, the SPCEs modified with AgNPs/rGO (blue line) display a sharp oxidation peak current due to the oxidation of AgNPs (Ag^0^ to Ag^+^) at 0.114 V and a small reduction peak located at −0.036 V. Approximately 6-fold higher intensity of oxidation response is observed. In addition, the overall background current is expanded for AgNPs/rGO due to the capacitive properties of rGO (Nossol et al., 2014). This suggests stripping of Ag^+^ from the AgNPs/rGO-modified electrode surface. The surface coverage or amount of Ag metal on the electrode is calculated to be 69.6 pmol cm^−2^ (7.51 ng cm ^−2^ Ag) (Fleming and Bond, 2009). The stripping response of AgNPs is employed for signal amplification of the label-free electrochemical immunosensor. The CV results of the three electrodes in 0.1 M PBS (pH 7.4) containing 5 mMK_3_ [Fe(CN)_6_]/K_4_[Fe(CN)_6_] in the scan range of potential from −0.4 to +0.8 V are also recorded, as shown in Figure 3A. All SPCEs display observable reversible couple peaks over the potential range. The cathodic (*I*_cp_) and anodic (*I*_ap_) peak currents and lowest peak-to-peak (Δ*E*_P_) are sequentially improved by modification using GO and AgNPs/rGO. The highest peak currents and the lowest Δ*E*_P_ value (0.3 V) are observed with AgNPs/rGO-modified SPCE, indicating the fastest electron transfer kinetics; while bare and GO-modified electrodes show Δ*E*_P_ values of 0.475 and 0.35 V, respectively. The SPCE modified with GO has higher current peaks than the bare electrode, caused by good electrochemical properties of the GO synthesized in the laboratory that we used (Jumpathong et al., 2016; Norfun et al., 2016). The AgNPs/rGO-modified SPCE presents the best electrochemical reactivity due to good conductivity of the AgNPs/rGO nanocomposite (Han et al., 2017). Electrochemical impedance spectroscopy (EIS) measurements of the modified electrodes were performed using 0.10 M PB (pH 7.4) with 5 mMK_3_[Fe(CN)_6_]/K_4_[Fe(CN)_6_] in a range of frequency from 100 kHz to 100 mHz at 220 mV. As shown in Figure 3B, EIS spectra have a semicircular fragment and a linear fragment, which coincide with the electron transfer process at higher frequencies and the electron diffusion process at lower frequencies. The size of the semicircle represents charge transfer resistance (*R*_et_) (Gündogdu et al., 2017). It was found that a very large semicircle (4,228 Ω) is observed for naked SPCE, indicating high electron transfer resistance. When GO is modified onto the SPCE, the *R*_et_ value significantly decreases to 1,266 Ω, suggesting lower resistance. This implies that the presence of GO on the electrode surface can improve electrochemical reactivity and electroactive surface area (Jumpathong et al., 2016; Norfun et al., 2016). The rGO adorned with AgNPs offers the smallest semicircle (974 Ω), since AgNPs and rGO could increase conductivity and improve the surface area of the SPCE (Nossol et al., 2014). EIS results are consistent with those of CVs and confirm that good electron transport on the rGO sheets sitting on SPCE is indeed strengthened by the decorating AgNPs.

**Figure 4:**
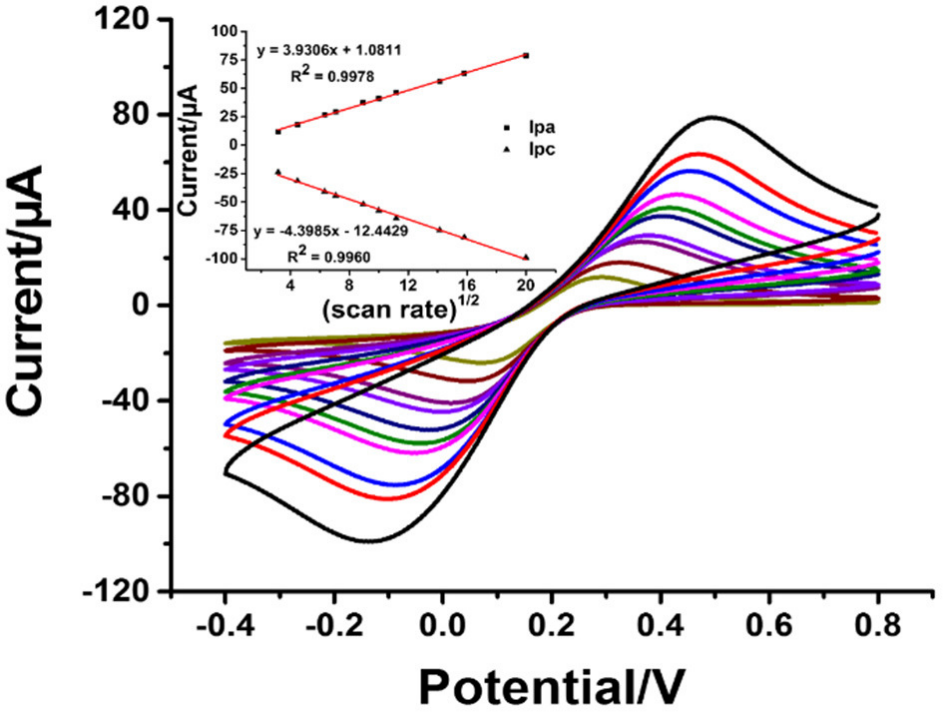
CVs of the AgNPs/GO-modified SPCE in contact with 0.010 M PB (pH 7.4) containing 5 mM K_3_[Fe(CN)_6_]/K_4_[Fe(CN)_6_] at different scan rates: 10, 20, 40, 50, 80, 100, 125, 200, 250, and 400 mVs^−1^. Inset: plots of the anodic peak current (*I*_pa_) and the cathodic peak current (*I*_pc_) vs. the square root of the scan rate.

To explain the electrochemical activity in the AgNPs/rGO-modified SPCE in contact with 0.10 M PB containing 5 mM of K_3_[Fe(CN)_6_]/K_4_[Fe(CN)_6_] as a model redox-active compound, CV results are recorded at different scan rates, as displayed in Figure 4. The optimized concentration of AgNPs/rGO of 1.5 mg.ml^−1^ is used in the electrode modification, referring to its highest oxidation and reduction peak currents. The anodic (*I*_pa_) and cathodic (*I*_pc_) peak currents display linearly relative to the square root of the scan rate (*v*^1/2^). The corresponding linear regression equations for both are found to be *I*_pa_ = 3.9306[*v*^1/2^] + 1.0811 (*R*^2^ = 0.9978) and *I*_pc_= −4.3985[*v*^1/2^] – 12.443 (*R*^2^ =0.996), respectively. This result indicates a diffusion-controlled process of electroactive species in the AgNPs/rGO-modified electrode (Chaiyo et al., 2016; Gao et al., 2016).

### Construction and Performance of Immunosensor Based on AgNPs/rGO Composite

**Figure 5:**
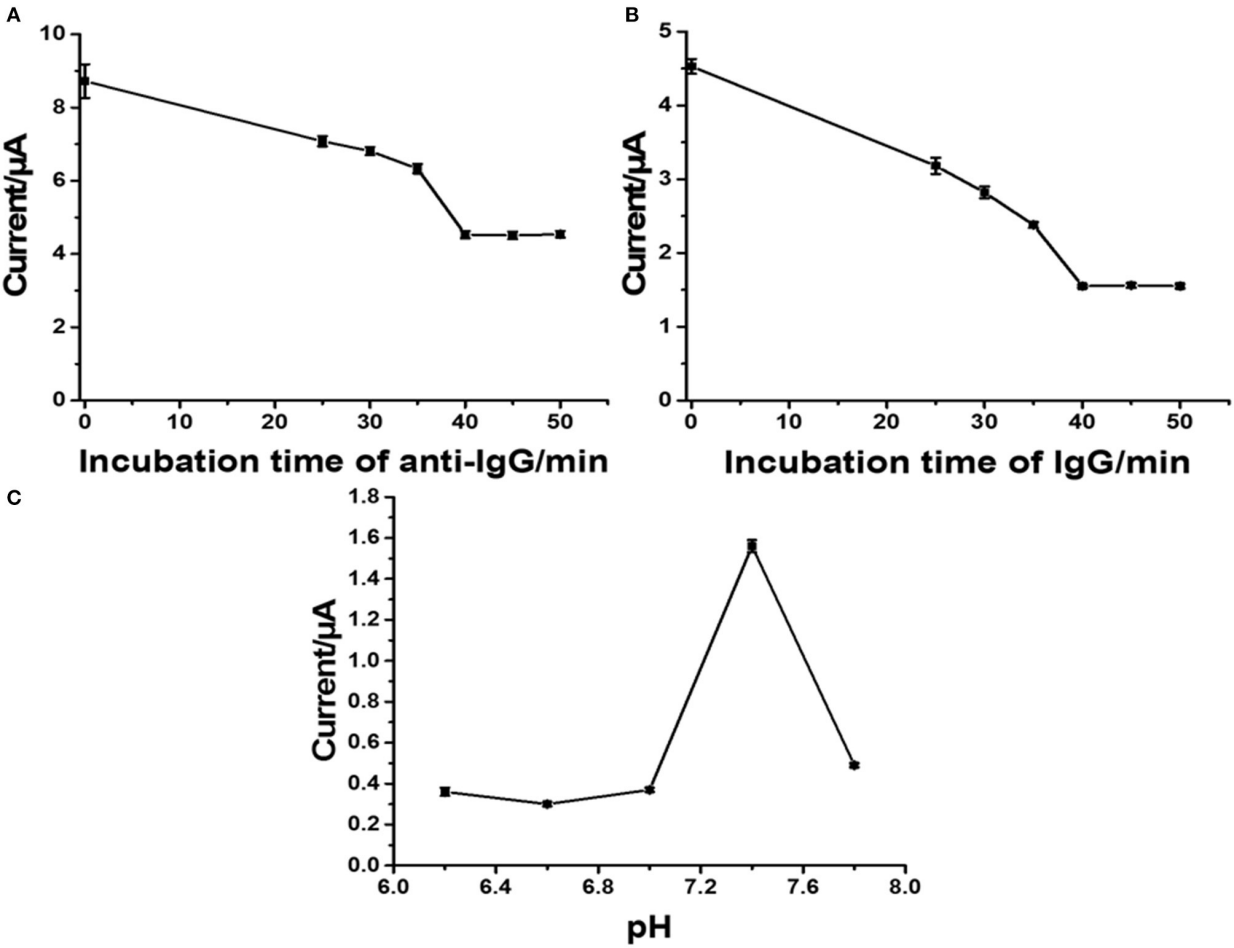
Influence of incubation time of anti-IgG **(A)**, incubation time of IgG **(B)**, pH of phosphate buffer **(C)** on current responses of the developed immunosensor.

With the employment of the anti-IgG antibody concentration of 50 mg.ml^−1^, the effect of incubation time on the immobilization of anti-IgG antibody was investigated from 25 to 50 min. A decrease in the stripping response of Ag was monitored, and the decrease is due to the blockage of immobilized antibodies. SWV results were recorded at a potential from 0 to 0.35 V (vs. Ag/AgCl) in 0.1 M PB (pH 7.4) with a pulse period of 2 s and an amplitude of 25 mV. Figure 5A shows the decrease in current signals (from ca. 8.8 to ca. 4.5 μA) when the incubation time was increased. At an incubation time of 40 min, the current remains constant (ca. 4.5 μA), indicating saturation of the antibodies on the electrode surface. From this point, immobilization does not further develop. Consequently, the incubation time of 40 min is used for the next optimization. Figure 5B demonstrates a study of incubation time for a complete reaction between surface-bound antibodies and target antigens when the electrode is incubated with the antigen solution (1 ng.ml^−1^ IgG). The reduction in peak current is due to the amount of immunocomplexes on the electrode surface. The reaction reaches an equilibrium point at an incubation time of 40 min, as seen with a plateau (ca. 1.5 μA). This indicates that from this incubation time immunoreaction cannot further occur. Therefore, the incubation time of 40 min is chosen for completion of the reaction of antigen-antibody. The pH value of an operating medium for detection of target is another important parameter, and it significantly affects the performance of an immunosensor in terms of stability and reproducibility, since the properties of antibodies depend on the environment. High acidity and basicity would cause denaturation of immobilized antibodies. Thus, the effect of pH value of the 0.10 M phosphate buffer (PB) on the current response of Ag in the immunosensor after binding with target antigens is studied at the pH range from 6.2 to 7.8, as shown in Figure 5C. It is found that the current response strongly depends on pH. The highest peak current is observed at the pH value of 7.4, while lower and higher pH values (<7.4 and >7.4) offer significant low current responses (ca. 35 and 0.5, respectively). This indicates that pH 7.4 would preserve the activity of the antibodies and immunocomplex. Therefore, pH is controlled at 7.4 for the rest of the measurement experiments (Duangkaew et al., 2015; Gao et al., 2016).

**Figure 6:**
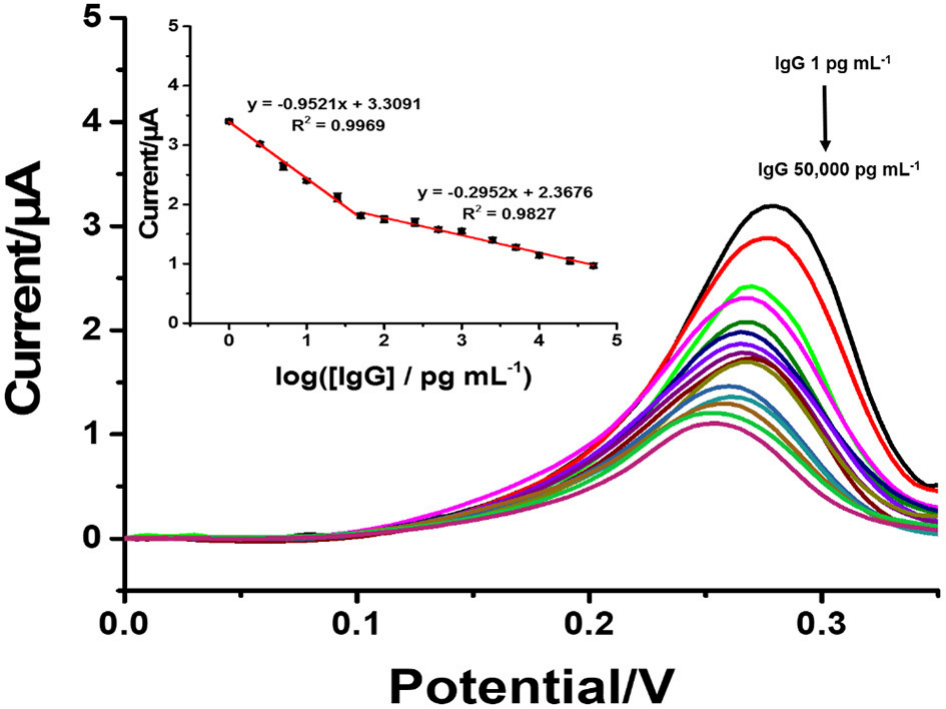
SWVs of the immunosensor after incubation with different concentrations of IgG. Inset: a calibration curve based on the change in the SWV peak current vs. the logarithm of the concentration (*n* = 3).

Under the optimal conditions of immunosensor mentioned above, current responses and the calibration curve (inset) for the determination of IgG are presented in Figure 6. The immunosensor displays decreased peak currents systematically after the antigen-antibody complex forms by incubation of immunosensing electrode with different IgG concentrations. It shows that the peak current decreases when the concentration of IgG increases. The reason for this is that the non-conductive antigen-antibody immunocomplex on the electrode surface acts as a blockage layer, which obstructs the electron transfer toward the electrode surface (Jumpathong et al., 2016; Norfun et al., 2016; Putnin et al., 2018). In this study, it would restrict the voltammetric stripping process of Ag+ from rGO sitting on the SPCE surface. Moreover, the result shows two good linear ranges between the reduced peak current and logarithm of target IgG concentration from 1 to 50 pg ml^−1^ and from 50 to 5 × 105 pg ml^−1^ with two regression equations, i.e., *I*_pa_ = −0.922 log[IgG] + 3.3631 (*R*^2^ = 0.9929) and *I*_pc_ = −0.2953 log[IgG] + 2.3676 (*R*^2^ = 0.9827) (where [IgG] represents the IgG concentration), and detection limit of 0.8608 pg ml^−1^. To further clarify the advantages of the proposed label-free electrochemical immunosensor, as shown in Table 1, we compare the analytical performance of the sensor with those of other label-free immunosensors toward detection of IgG with respect to detection range and LOD. In this study, linear dynamic range is demonstrated with a comparable wide range, while LOD is much lower than those from other immunosensors. The immunosensor finds its simplicity in detection and operation with not having to use external redox probes for signal amplification, ease in scalable material production, and consumption of inexpensive reagents or precursors. This table confirms that the performances of the proposed immunosensor are acceptable.

**Table 1:** Comparison of our proposed immunosensor with other reported sensors in the determination of IgG.

Modified electrode^*^	**Detection** method^*^	**Linear range** **(ng.ml^−1^)**	**Detection limit** **(ng.ml^−1^)**	**References**
GO/SPCE	Amp	2–100	1.70	Thunkhamrak et al., 2017
Chit/CNT-IL/AuE	DPV	0.1–15	0.02	Shen and Shen, 2019
PtNPs/PAAMI/LAG	DPV	0.012–352	0.006	Barman et al., 2020
GO/PAA/SPCE	DPV	1–100	0.54	Norfun et al., 2016
GO/SPCE	DPV	2.5–100	1.99	Jumpathong et al., 2016
P2ABA/SPCE	DPV	1–50	0.5	Putnin et al., 2018
PP3C/SPCE	DPV	0.50–50	0.12	Chanarsa et al., 2020
AMPPH-AuNPs/GCE	DPV	0.1–5 and 5–100	0.08	Li R. et al., 2014
TAA/AuE	DPV	0.01–25	0.003	Shen et al., 2015
rGO-Ag NPs/SPCE	SWV	0.001–0.05 and 0.05–50	0.00086	This work

* *GO, graphene oxide; SPCE, screen printed carbon electrode; Amp, amperometry; Chit, chitosan; CNT-IL, ionic liquid functionalized carbon nanotube; AuE, gold electrode; DPV, differential pulse voltammetry; PtNPs, platinum nanoparticles; PAAMI, polyelectrolyte polyallylamine; LAG, laser-ablated graphene; PAA, poly(acrylic acid); P2ABA, poly(2-aminobenzylamine); PP3C, poly(pyrrole-3-carboxylic acid); AMPPH, 4-amino-1-(3-mercapto-propyl)-pyridine hexafluorophosphate; AuNPs, gold nanoparticles; GCE, glassy carbon electrode; TAA, thiol aromatic aldehyde; rGO, reduced graphene oxide; Ag NPs, silver nanoparticles; SWV, square wave voltammetry*.

**Figure 7:**
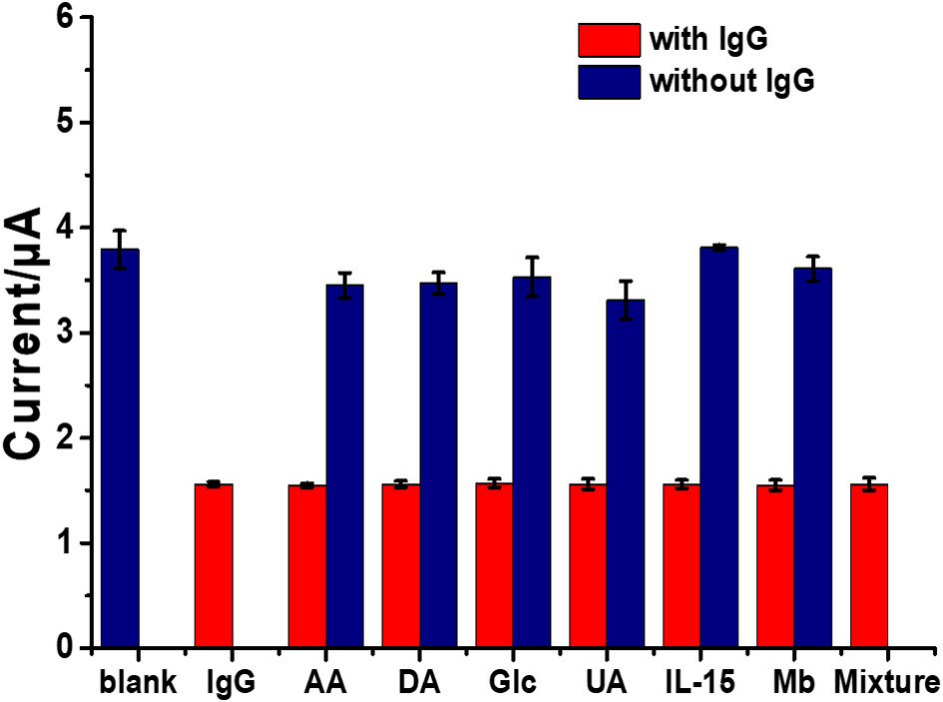
Selectivity study of the immunosensors incubated with different solutions: blank (blue bar), IgG solution (1 ng ml^−1^, red bar), interference solutions (100 ng ml^−1^) with presence of 1 ng ml^−1^ IgG (red bars), and individual interference solutions (100 ng ml^−1^) with no IgG (blue bars).

Selectivity, reproducibility, and stability are important parameters in terms of construction and applicability of immunosensors. Moreover, selectivity of an immunosensor has a significant role in the detection of a target in a sample without separation. To examine the selectivity of the sensor, the surfaces of the immunosensors were incubated in blank solution, in individual interference solutions, and in different IgG solutions without and with the presence of individual and mixed interfering agents. Interference experiments were performed using an IgG target antigen solution (1 ng ml^−1^) with ascorbic acid (AA), dopamine (DA), glucose (Glu), uric acid (UA), interleukin-15 (IL-15), and myoglobin (Mb) or their mixture (100 ng ml^−1^) together with the solutions of these interferences with no IgG. As shown in Figure 7, it is found that the peak current responses of the immunosensor are reduced (ca. 1.55 μA) when it is incubated with solutions containing IgG. At the IgG concentration of 1 ng ml^−1^, the decreases in the signal are significantly different from that of blank, remaining ~59% of the initial response. The peak current responses (a range of ca. 3.35–3.8 μA) of individual interference solutions (100 ng ml^−1^ AA, DA, Glu, UA, IL-15, or Mb) are different from that of the blank solution (ca. 3.78 μA). Reduced current responses when the immunosensor was incubated with the solution containing IgG (including the mixture with IgG) have no significant difference among each other. This manifests high selectivity compared with all the interferences. The results indicated that the immunosensor exhibited excellent selectivity for the detection of IgG (Jumpathong et al., 2016; Li et al., 2017). The selectivity, reproducibility, and stability of the immunosensor based on an AgNPs/rGO-modified SPCE are all acceptable; therefore, it has the ability for quantitative detection of IgG in real samples from humans. To evaluate reproducibility, 18 individual immunosensors based on an AgNPs/rGO modified SPCE were constructed by the same process. For incubation with the blank solution (Supplementary Figure 2), the average current response and the relative standard deviation (RSD) from nine individual sensors were observed to be 3.74 μA and 1.45%, respectively. Moreover, in the detection of IgG at a concentration of 1 ng ml^−1^ by the other nine immunosensors, the average current response and the %RSD value were 1.55 μA and 1.22%, respectively, suggesting acceptable precision and reproducibility of the proposed label-free electrochemical immunosensor. Additionally, stability was evaluated by storing the immunosensor at 4°C prior to testing. After storage for 14 days, the immunosensor retains 97.63% of its initial current signal for detection of IgG at a concentration of 1 ng ml^−1^, as shown in Supplementary Figure 3, indicating that the immunosensor possesses good stability.

A series of human serum samples with known amounts of spiked human IgG concentrations was analyzed to evaluate the possibility of the suggested immunoassay, as shown in Table 2. Prior to the assay, the serum samples with a high level of analyte were suitably diluted with 0.1 M PB (pH 7.4). The % recoveries and %RSDs were found to be in the ranges of 89.19–109.65% and 1.13–3.3%, respectively, suggesting acceptable performance of the immunosensor. It is plausible that it is a great promising device for the detection of IgG in real sample analysis (Wu et al., 2013; Huang et al., 2015).

**Table 2:** Recovery study of the prepared immunosensor.

**Sample**	**Standard of IgG (pg.ml^−1^)**	**Found (pg.ml^−1^)**	**Recovery (%)**	**RSD (%)**
1	1.0	1.10	109.65	1.35
2	2.5	2.24	89.64	1.79
3	5.0	5.24	104.77	1.13
4	10	10.54	105.41	1.19
5	25	22.30	89.19	3.30

## Conclusion

In this study, we have successfully constructed a label-free electrochemical immunosensor using an AgNPs/rGO composite-modified SPCE for the determination of IgG. The AgNPs/rGO composite with excellent potential for use as a signal amplifier in the electrochemical immunosensor can improve the electrochemical reactivity of SPCE. Under conditional optimization, our label-free electrochemical immunosensor presents high sensitivity, two wide linear calibration ranges from 1 to 50 pg.ml^−1^ and 50 to 5 × 10^5^ pg.ml^−1^, and a low LOD value of 0.86 pg ml ^−1^. Furthermore, the fabricated label-free electrochemical immunosensor exhibits high specificity to IgG with the presence of 100-fold interfering substances. The advantages of this label-free electrochemical immunosensor include simple preparation, high sensitivity, high selectivity, and low cost. The immunosensor could be further applied for clinical diagnosis and developed for the detection of other protein biomarkers.

## Data Availability Statement

The original contributions presented in the study are included in the article/Supplementary Material, further inquiries can be directed to the corresponding author/s.

## Author Contributions

SC: investigation and data curation. JJ: writing—review and editing. KO: conceptualization, methodology, formal analysis, resources, validation, writing—original draft, writing—review, editing, supervision, project administration, and fund acquisition. All authors contributed to the article and approved the submitted version.

## Conflict of Interest

The authors declare that the research was conducted in the absence of any commercial or financial relationships that could be construed as a potential conflict of interest.
