# Supplementary material for: A Bifunctional Nanosilver-Reduced Graphene Oxide Nanocomposite for Label-Free Electrochemical Immunosensing
Source: Front Chem. 2021 Apr 28;9:631571. doi: 10.3389/fchem.2021.631571 (PMC8113703; doi:10.3389/fchem.2021.631571)
Supplement: Supplementary file 1 [file Data_Sheet_1.PDF]

Supplementary Material

A Bifunctional Nanosilver-Reduced Graphene Oxide Nanocomposite for Label-Free Electrochemical Immunosensing

Supakeit Chanarsa[1,2], Jaroon Jakmunee[1,3], Kontad Ounnunkad[1,3,4*]

[1]Department of Chemistry and Center of Excellence for Innovation in Chemistry, Faculty of Science, Chiang Mai University, Chiang Mai 50200, Thailand

[2]The Graduate School, Chiang Mai University, Chiang Mai 50200, Thailand

[3]Research Center on Chemistry for Development of Health Promoting Products from Northern Resources, Chiang Mai University, Chiang Mai, 50200, Thailand

[4]Center of Excellence in Materials Science and Technology, Chiang Mai University, Chiang Mai 50200, Thailand

*** Correspondence:**
Corresponding Author
kontad.ounnunkad@cmu.ac.th
suriyacmu@yahoo.com

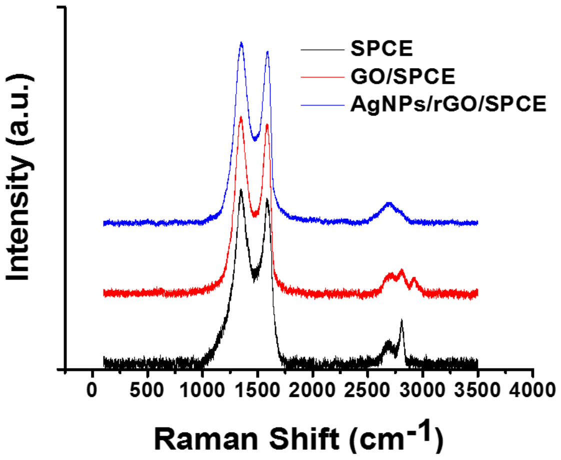

Figure S1 Raman spectra of bare SPCE and GO- and AgNPs/rGO-modified SPCEs.

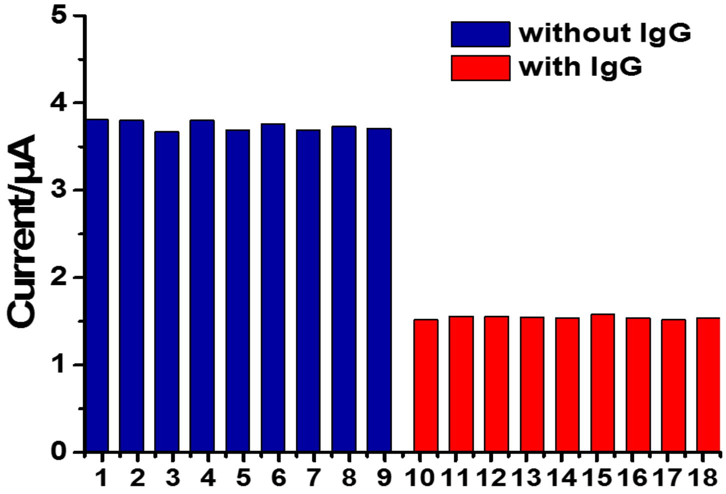

Figure S2 Reproducibility study of immunosensor for detection of IgG in blank solution and in IgG solution at a concentration of 1.0 ng mL^{-1}.

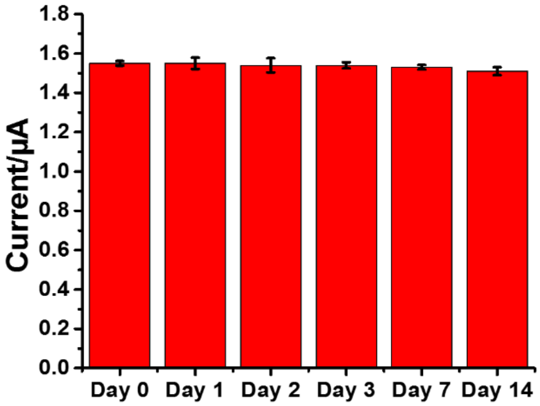

Figure S3 The current responses of the immunosensor for detection of 1.0 ng mL^{-1} IgG after storage for 0, 1, 2, 3, 7, and 14 days.
